# Investigating the effectiveness and cost-effectiveness of FITNET-NHS (Fatigue In Teenagers on the interNET in the NHS) compared to activity management to treat paediatric chronic fatigue syndrome (CFS)/myalgic encephalomyelitis (ME): amendment to the published protocol

**DOI:** 10.1186/s13063-019-3895-1

**Published:** 2019-12-19

**Authors:** Emma Anderson, Daisy Gaunt, Chris Metcalfe, Manmita Rai, William Hollingworth, Nicola Mills, Lucy Beasant, Roxanne Parslow, David Kessler, John Macleod, Paul Stallard, Hans Knoop, Elise Van de Putte, Sanne Nijhof, Gijs Bleijenberg, Esther Crawley

**Affiliations:** 1 Centre for Child Health, Bristol Medical School: Population Health Sciences, University of Bristol, 1-5 Whiteladies Road, Bristol, BS8 1NU UK; 2 Bristol Medical School: Population Health Sciences, University of Bristol, Canynge Hall, 39 Whatley Road, Bristol, BS8 2PS UK; 3 Bristol Randomised Trials Collaboration, University of Bristol, Bristol, UK; 4 Bristol Medical School: Population Health Sciences, Oakfield House, Oakfield Grove, Bristol, BS8 2BN UK; 5 Department for Health, University of Bath, Bath, BA2 7AD UK; 6 Department for Medical Psychology, University Medical Centres Amsterdam, University of Amsterdam, Postbox 22660, 1100 DD Amsterdam, The Netherlands; 7 Department of Paediatrics, Wilhelmina Children’s Hospital, University Medical Centre, Utrecht, The Netherlands; 8 Radboud University Medical Center, Nijmegen, The Netherlands

**Keywords:** Paediatrics, Chronic fatigue syndrome, Myalgic encephalomyelitis, CFS/ME, CBT, Online systems, E-therapy, Methodology, Recruitment, Trial

## Abstract

**Abstract:**

The FITNET-NHS Trial is a UK, national, trial investigating whether an online cognitive behavioural therapy program (FITNET-NHS) for treating chronic fatigue syndrome/ME in adolescents is clinically effective and cost-effective in the NHS. At the time of writing (September 2019), the trial was recruiting participants. This article presents an update to the planned sample size and data collection duration previously published within the trial protocol.

**Trial registration:**

ISRCTN, ID: 18020851. Registered 8 April 2016.

## Background

The FITNET-NHS Trial is a United Kingdom (UK), national ,randomised controlled trial testing the acceptability, effectiveness and cost-effectiveness of an online cognitive behavioural therapy (CBT) programme, FITNET-NHS (Fatigue In Teenagers on the interNET in the National Health Service), designed to treat adolescents with chronic fatigue syndrome/myalgic encephalomyelitis (CFS/ME) [1]. In the UK, most young people with CFS/ME do not have access to local NHS specialist medical care for the condition, as there are limited paediatric CFS/ME specialist centres in the UK. The FITNET-NHS trial was set up to test the *online delivery* of specialist care from one specialist paediatric CFS/ME site in the south west of England as a means of addressing this problem. Recruitment projections were estimated in advance and were based on reaching national paediatric CFS/ME populations at high volumes – made possible due to our innovative methodology using entirely remote processes for recruitment and treatment delivery. The original sample size target if achieved would have given us sufficient statistical power to detect a true effect of FITNET-NHS within the subgroup of participants with co-morbid mood disorder.

The current article is an amendment to our previously published protocol for the FITNET-NHS Trial: Baos et al. (2018) [1].

## Amendment

Recruitment into the FITNET-NHS Trial began on 1 November 2016. In mid-2018, it became apparent that the recruitment rate would not allow us to achieve our original sample size target (*n* = 734) without a substantial extension to the recruitment period. The original sample size was selected to provide 80% power to detect a 0.4-standard deviation (SD) difference at 5% significance with 10% attrition on the primary outcome in a *subgroup* of participants (estimated to be 30% or *n* = 220) with co-morbid mood disorders of anxiety and depression. For all participants (with or without co-morbid mood disorders), the original sample size provided 97% power at 1% significance to detect a 0.35-SD difference on the primary outcome (Short Form Health Survey; Physical function Subscale (SF-36-PFS) score) at 6 months.

We reviewed the trial with the funders – National Institute for Health Research, Health Technology Assessment (NIHR HTA, on 10 July 2018) and consulted with the Trial Management Group (TMG, on 12 September 2018 and 18 October 2018), the Data Safety Monitoring Committee (DSMC, on 10 October 2018 and by email report on 11 March 2019) and the Trial Steering Committee (TSC, on 28 November 2018).

In September 2018, we calculated the required sample size for the primary outcome in all participants (with or without co-morbid mood disorders):

Data on 266 children will give us 90% power at 5% significance to detect a 0.4-SD difference on the SF-36-PFS. With attrition currently at approximately 15%, we will need to recruit 314 children. This is achievable (based on recruitment rates to date) by the end of October 2020.

### This gave us a new recruitment target of 314 children in total, 157 in each treatment group

We considered the issue of co-morbid disorders. On 2 October 2018, we investigated the rate of co-morbid mood disorders in FITNET-NHS participants at baseline. This was higher than our original estimates as the rate of co-morbid mood disorders was 40% (compared to 30% in our original estimates). With the revised sample size target of 314 there will be approximately 106 participants with co-morbid mood disorders (53 in each treatment group). This will give 53% power at 5% significance to detect a 0.4-SD difference on the SF-36-PFS between treatment groups within this co-morbid subgroup.

From consultation with the NIHR HTA, TSC, DSMC and TMG, the decision was made to agree revised recruitment targets and extend the recruitment time by 6 months to enable the FITNET-NHS Trial to achieve the primary aim of testing the effectiveness (and cost-effectiveness) of the FITNET-NHS treatment compared to Activity Management. The NIHR HTA gave their approval in principle of this change on 30 January 2019, subsequent to provision of documentation. Full NIHR HTA approval for revised recruitment target and the contract variation to include the 6-month extension to the project timeline was received on 24 April 2019. We will, therefore, recruit 314 children and recruitment will finish on 31 October 2020. Follow up will finish on 31 October 2021.

We have followed standard procedures to update all relevant organisations regarding these changes, including trial registration (ISRCTN, ID: 18020851: change accepted and records updated on 1 July 2019), and submitting as a substantial trial amendment to the Research Ethics Committee (REC, approved on 26 July 2019) and the Health Research Authority (HRA, approved on 10 July 2019).

With our revised recruitment target of 314 participants, the FITNET-NHS is still set to be the largest paediatric CFS/ME treatment trial in the UK and globally, the results of which will should the future of paediatric CFS/ME treatment delivery.

## Data Availability

The research staff will have access to the de-identified data during the project period, and 5 years following the completion of the trial for contributing to knowledge building through dissemination of research reports.

## Abbreviations

CBT: Cognitive behavioural therapy
CFS/ME: Chronic fatigue syndrome/myalgic encephalomyelitis
DSMC: Data Safety and Monitoring Committee
FITNET-NHS: Fatigue In Teenagers on the interNET in the NHS
HRA: Health Research Authority
ISRCTN: International Standard Randomised Controlled Trials Number
NIHR HTA: National Institute for Health Research, Health Technology Assessment
REC: Research Ethics Committee
SD: Standard deviation
SF-36-PFS: Short Form Health Survey (36 question): Physical Function Subscale
TMG: Trial Management Group
TSC: Trial Steering Committee
UK: United Kingdom
